# Prevalence of gastrointestinal nematodes and *Fasciola hepatica* in sheep in the northwest of Spain: relation to climatic conditions and/or man-made environmental modifications

**DOI:** 10.1186/1756-3305-6-282

**Published:** 2013-09-27

**Authors:** María Martínez-Valladares, David Robles-Pérez, Jose Manuel Martínez-Pérez, Coral Cordero-Pérez, Ma del Rosario Famularo, Nélida Fernández-Pato, Camino González-Lanza, Luciano Castañón-Ordóñez, Francisco A Rojo-Vázquez

**Affiliations:** 1 Instituto de Ganadería de Montana, CSIC-ULE, Finca de Marzanas, 24346, Grulleros León, Spain; 2 Faculty of Veterinary Medicine, University of León, Campus de Vegazana, 24071 León, Spain

**Keywords:** Gastrointestinal nematodes, Fasciola hepatica, Sheep, Prevalence, Climatic conditions

## Abstract

**Background:**

In the present study we studied and updated the prevalence of the infections caused by gastrointestinal nematodes (GIN) and *Fasciola hepatica* in grazing sheep in the northwest (NW) of Spain for the last six years (2006–2011), and its relationship with the current climatic conditions.

**Methods:**

We analyzed faecal samples from 110 flocks located in four different provinces of the Autonomous Community of Castilla y León: 76.4% of them were situated in León, 12.7% in Zamora, 9.1% in Palencia and 1.8% in Valladolid.

**Results:**

The prevalence of GIN was 100% and the mean of eggs per gram (epg) in faeces was 237.2 (± 375.9) per flock. Regarding climatic conditions, we found a direct relationship between the GIN infection level and the maximum humidity (p<0.05) but inverse with the degree of solar radiation (p<0.05). The prevalence of fasciolosis was 59.3%, with a mean epg of 17.5 (± 33.9) per flock; these values were correlated with the minimum humidity and precipitations (p<0.05). Comparing our results in León with previous studies during the early 1990s, the mean epg of GIN was increased slightly (134.3 epg); regarding fasciolosis, the prevalence rose significantly, from 26.7% to 60.5%. Since the 1990s we observed that the maximum temperature is nowadays 0.45°C higher (17.0°C) and the minimum 0.5°C lower (5.2°C); the rainfall values were very similar in both decades but at the present time the humidity is higher (75.9%).

**Conclusions:**

We found that the prevalence of GIN and *F. hepatica* infections was directly influenced by the humidity and also by precipitations in the case of *F. hepatica*. Comparing the current prevalence with studies carried out in the same area for the early 1990s, we observed that nowadays the mean epg of GIN is higher with a possible cause being the differences in climatic conditions depending on the sampling year. Regarding *F. hepatica* infection, its prevalence rose significantly probably favoured by an increase in irrigated areas in the area of study.

## Background

The infections caused by helminth parasites are very common in ruminants worldwide but principally they are present in areas with enough humidity for the development of free-living stages of the life cycle. The prevalence of some of them can reach 100% in those flocks with grazing animals. The importance of helminth infections is due to the economic losses related with lower growth and milk production as well as liver condemnations. Groups of importance for sheep are the nematodes and trematodes (the most prevalent in Europe, America and Australia), and also the cestodes. Their life cycles include free-living stages directly influenced by the climate, - principally temperature, rainfalls and humidity - regulating the geographical distribution of some of the species. Recent data suggest that the pattern of helminth infections may be changing in Scotland since the climate in the United Kingdom is varying. Van Dijk *et al.*[1] described that, over the past 5–10 years, highly significant increases in the overall rate of diagnosis of gastrointestinal nematodes (GIN) were observed in Great Britain and suggested that the effect of climate change on parasite epidemiology proved to be the most likely explanation for the observed patterns. In fact, there are some reports of production-limiting disease outbreaks caused by GIN such as *Haemonchus contortus*, *Nematodirus battus*, *Teladorsagia circumcincta* and also by *Fasciola hepatica* in sheep flocks in the south-eastern of Scotland [2]. In the Netherlands, the summer of 2003 was unusually warm and dry and the consequence was that the proportion of GIN eggs that developed into infective larvae was very low, although severe *H. contortus* infections were observed in lambs during that period [3].

In this context, the objective of the present study was to update the prevalence of helminth infections, GIN and *F. hepatica*, in grazing sheep in the north-west (NW) of Spain between the years 2006 and 2011. Moreover, we determined their relationship with the current climatic conditions and established the joint evolution of climate and helminth prevalence in the last two decades after comparing our results with previous studies carried out in the same sampling area [4,5].

## Methods

### Description of sampling area

**Figure 1:**
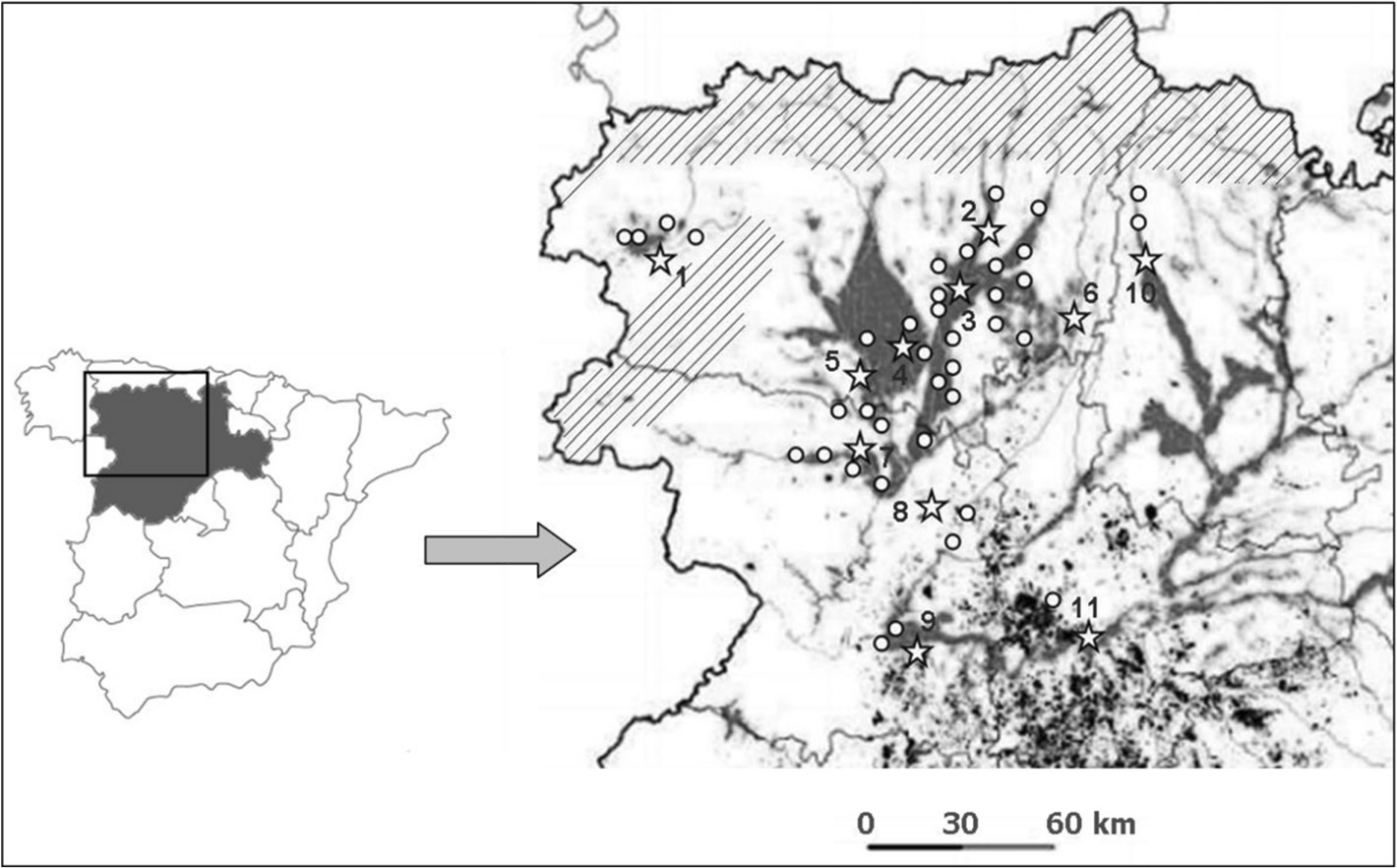
**Map of the autonomous community of Castilla y León (Spain).** The stars show the meteorological stations and the white points the towns/villages where the farms sampled were situated. The rivers and irrigated areas (dark areas) are shown in the map as well as the mountain range (lines).

The study was carried out for 6 years, from January 2006 until December 2011, in sheep flocks located in the Autonomous Community of Castilla y León, NW of Spain, a region constituted by nine provinces. The sampled area was situated mainly in four provinces: León, Zamora, Palencia and Valladolid but most of the flocks were located in León (Figure 1).

The sampling areas in the four provinces have similar climatic conditions, a Mediterranean climate with continental and Atlantic influences, characterized by cold winters and warm summers. The altitude of the sampling area within the Autonomous Community (average altitude of about 800 metres) and its mountain ranges contributes not only to the difference between summer and winter temperatures, but also to a marked contrast between day and night temperatures. Winters are long and cold, with average temperatures between 4°C and 7°C in January and summers are short and hot, with averages between 19°C and 22°C. On the other hand, the rainfall in this community is not very high, with total annual values between 347 mm^3^ in the province of Palencia and 531 mm^3^ in León.

### Sheep flock description

Most of the sheep flocks were sampled from October until the end of June. Different sheep breeds can be found depending on the production and management systems. Flocks under intensive management and mainly kept for milk production have Assaf breed sheep; the Churra and Merino breed flocks are mainly raised for meat under a semi-extensive or extensive systems in which animals graze on pastures for 6–8 hours per day and the rest of the time are indoors.

All sampled sheep were older than 1 year old and were not pregnant at the sampling moment with the aim to avoid the influence of host immunity on the faecal egg count.

Flocks included in this survey grazed at the time of the study. Moreover, the yearly sampling was always carried out before any anthelmintic administration and at least 2 months after the last treatment. Sheep were usually treated in spring and/or autumn with albendazole, levamisole or ivermectin to control GIN and with albendazole, triclabendazole or clorsulon in those farms with previous fasciolosis diagnosis.

A total of 110 sheep flocks were studied in the following provinces: 84 in León, 14 in Zamora, 10 in Palencia and 2 in Valladolid.

### Climatic conditions

The climatic conditions of the localities where farms were situated were registered with the aim to correlate them with the prevalence data of GIN and *F. hepatica*. Since the prepatent period for GIN is around 2–3 weeks and between 8–12 for *F. hepatica*, the climate data were collected for a period of 30 days before the prepatency, when the free-living stages are developing in the field. Due to this reason we calculated the average of the registered data between the days 15 and 45 before taking the faecal samples for GIN, and between the days 60 and 90 for *F. hepatica*.

Climate data of each farm were registered for the period of 30 days before the prepatency, as it is described previously, from the nearest meteorological station (http://www.inforiego.org) (Figure 1 and Table 1). The data collected were: average temperature (°C), maximum temperature (°C), minimum temperature (°C), average humidity (%), maximum humidity (%), minimum humidity (%), solar radiation (MJ/m^2^) and precipitation (mm^3^). All these climate variables were correlated with the prevalence data of GIN and *F. hepatica*.

**Table 1:** Coordinates and altitude of meteorological stations

**Meteorological station**	**Coordinates**	**Altitude (m.a.s.l.)**
1	42° 33′ 17″ N, 6° 44′ 2″ W	456
2	42° 30′ 32″ N, 5° 26′ 31″ W	790
3	42° 22′ 22″ N, 5° 30′ 27″ W	777
4	42° 16′ 19″ N, 5° 44′ 9″ W	783
5	42° 12′ 22″ N, 5° 51′ 4″ W	746
6	42° 22′ 19″ N, 5° 1′ 49″ W	828
7	42° 00′ 13.4″ N, 5° 48′ 34.8″ W	719
8	41° 56′ 3″ N, 5° 39′ 51″ W	712
9	41° 29′ 32″ N, 5° 41′ 5″ W	711
10	42° 31′ 26″ N, 4° 45′ 55″ W	916
11	41° 52′ 59″ N, 5° 2′ 34″ W	733
12	41° 24′ 50″ N, 4° 57′ 35″ W	728

In the current study we also compare our prevalence data (2006–2011) in the province of León with studies carried out by other authors previously in the same area. Regarding GIN, we compared the current prevalence with the results obtained by Martínez-González *et al.*[5] during 1991–1992; the annual climatic conditions for this period of time were also registered. In relation to fasciolosis, we contrasted the results obtained in the province of León with the study carried out by Ferre *et al.*[4] in the same area. We analysed the climatic conditions during the sampling period carried out by these authors, between October 1992 and May 1993. With the aim to compare all these studies, the mean monthly climate data in the province of León were obtained from the website http://www.tutiempo.net and were: average temperature (°C), maximum temperature (°C), minimum temperature (°C), average humidity (%) and annual precipitations (mm^3^).

### Sample collection and GIN species identification

The selection of the flocks was made by taking 20 individual faecal samples in each one and determining the faecal egg count for each sheep. Then, the mean of these samples were calculated for each flock. All individual samples were analysed twice, by sedimentation and flotation techniques, to detect GIN and *F. hepatica* eggs respectively and using a McMaster chamber for counting eggs. The sensitivity of both techniques was 15 eggs per gram (epg).

The GIN species were identified after faecal cultures of faeces [6]. The morphological study of at least 100 L3 per faecal culture was carried out following [7].

The protocol of the sample collection was approved by the ethics committee of the Instituto de Ganadería de Montaña (IGM, León. Spain).

### Statistical analysis

All eggs output in faeces were expressed as their mean (± standard deviation). The data were analysed using the statistical computer package for social science, SPSS. Faecal egg counts were transformed to Ln (X + 1) to produce approximately normally distributed data before calculating the correlations with the climatic variables by means of the Pearson’s coefficient. A parametric test for multiple comparisons, the One-way ANOVA was used to determine the significant differences between years. In order to determine whether or not there were significant differences between two years, a Student’s test was carried out. Differences below the 5% level (P < 0.05) were considered to be significant.

## Results

### Prevalence of gastrointestinal nematodes and its relation with climatic conditions

With the aim to detect the helminth infection status in ovines, 110 sheep farms were studied for six years (2006–2011) in some provinces of the Autonomous Community of Castilla y León (Spain): 76.4% of them were located in León, 12.7% in Zamora, 9.1% in Palencia and 1.8% in Valladolid.

**Figure 2:**
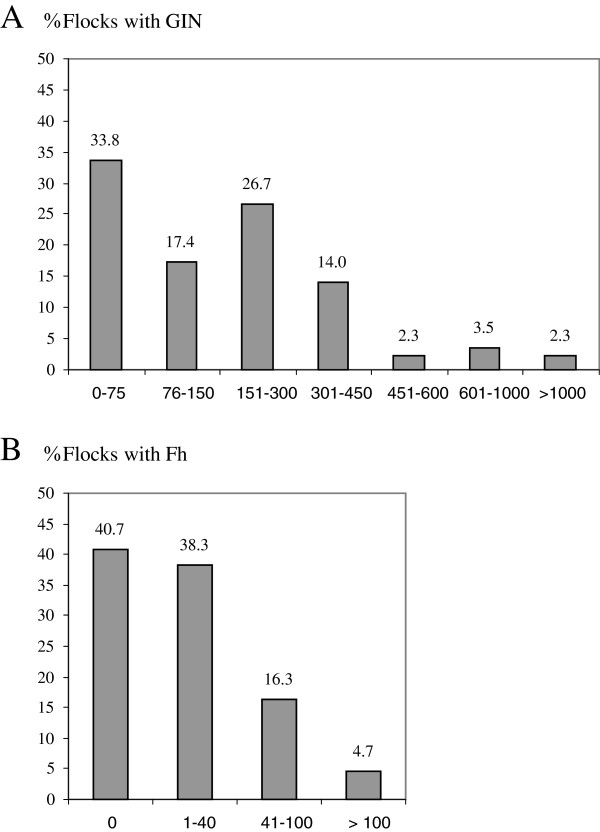
**Percentage of sampled flocks distributed according to their mean of GIN (2A) and *F. hepatica *epg values (2B).**

**Figure 3:**
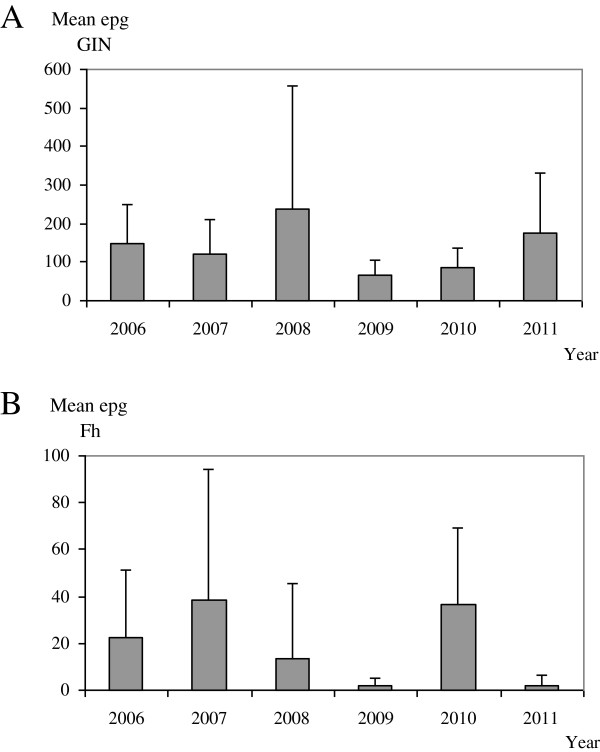
**Mean epg in flocks according to the sampled year in GIN (3A) and in *F. hepatica *(3B) infections.**

The prevalence of GIN infection was 100% and its distribution, according to the mean epg in each flock, is shown in the Figure 2A. The mean epg was 237.2 (± 375.9) per flock, with values between 5 and 2,552 epg. During the whole study, the level of infection in 51.2% of the farms sampled was low, with a mean per flock less than 150 epg. Nevertheless, 43% of the sampled flocks had high values of eggs output in faeces, with a mean between 150 and 600 epg; only 5.8% of them had a very high infection level with a mean higher than 600 epg. We did not find any significant differences in the epg values between the six years although the highest values were obtained in 2008 with a mean epg of 237.5 (± 320.4) (Figure 3A). For the rest of the years the mean values ranged between 66.7 (± 37.8) in 2009 and 175 (± 157.7) in 2011 (Figure 3A).

The most prevalent GIN species were *Teladorsagia circumcincta* (67–85%) followed by *Trichostrongylus* spp (7–21%), *Chabertia ovina* (0–22%), *Haemonchus contortus* (0–16%), *Bunostomum* spp (0–12%) and *Nematodirus* spp (0–3%).

**Figure 4:**
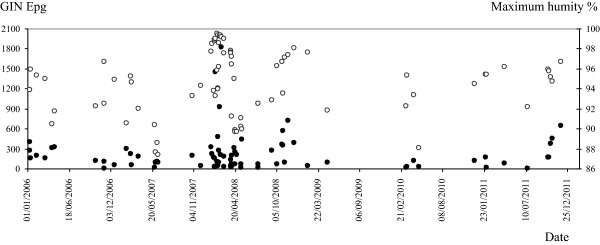
Distribution of the gastrointestinal nematode (GIN) eggs per gram (epg) in faeces along the six years of the study (black spots) in comparison to the maximum humidity (%) (white spots).

**Figure 5:**
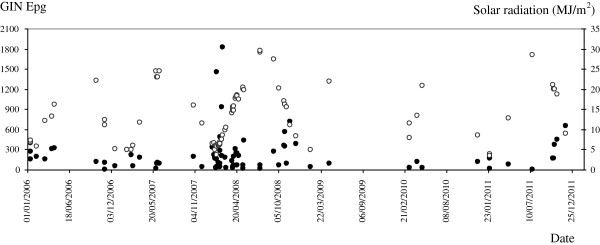
**Distribution of the gastrointestinal nematode (GIN) eggs per gram (epg) in faeces along the six years of the study (black spots) in comparison to the solar radiation (MJ/m**^**2**^**) (white spots).**

The GIN epg values are correlated with the climate data registered for 1 month before the prepatent period of the sampled faeces (Table 2). The Pearson’s correlation reflected that there is a direct significant relationship of the GIN infection level with the maximum humidity (p<0.05) but inverse with the degree of solar radiation (p<0.05). The distribution of the GIN epg in faeces along the study in comparison to the maximum humidity and solar radiation, the climate variables with which the highest correlations were shown, are represented in Figures 4 and 5, respectively.

**Table 2:** **Pearson correlation between GIN and *F. hepatica *epg values and climate data**

	**GIN epg**		***F. hepatica *epg**	
	**Correlation (r)**	**Signification (p)**	**Correlation (r)**	**Signification (p)**
**Average temperature (°C)**	−0.120	0.276	−0.011	0.930
**Maximum temperature (°C)**	−0.078	0.480	−0.023	0.853
**Minimum temperature (°C)**	−0.130	0.239	0.037	0.764
**Average humidity (%)**	0.175	0.056	0.175	0.065
**Maximum humidity (%)**	0.186	0.045*	0.045	0.349
**Minimum humidity (%)**	0.133	0.113	0.232	0.022*
**Solar radiation (MJ/m**^**2**^**)**	−0.185	0.046*	−0.146	0.105
**Precipitations (mm**^**3**^**)**	−0.015	0.447	0.235	0.020*

Significant correlations (p<0.05) are indicated with an asterisk (*).

In order to compare the current prevalence of GIN and climate data with previous results obtained by Martínez-González *et al.*[5] in the same sampling area, we registered the annual climatic conditions for the years 1991–1992 that are shown in Table 3.

**Table 3:** Annual climate data during the years 1991–1992 and 2006-2011

**Year**	**Average temperature (°C)**	**Maximum temperature (°C)**	**Minimum temperature (°C)**	**Average humidity (%)**	**Annual precipitations (mm**^**3**^**)**
1991	12.30	16.60	5.80	61.40	357.9
1992	12.20	16.50	5.60	61.00	357.6
Average	12.25	16.55	5.70	61.20	357.8
2006	13.30	17.50	6.40	63.40	573.6
2007	11.80	16.50	5.10	67.20	369.3
2008	11.40	16.60	4.90	65.90	393.2
2009	12.10	17.50	4.90	60.70	417.3
2010	10.90	16.20	4.60	65.80	552.9
2011	12.10	17.70	5.30	64.50	494.6
Average	11.93	17.00	5.20	64.58	466.8

### Prevalence of F. hepatica infection and its relation with climatic conditions

The prevalence of liver fluke infection in the farms included in the present study was 59.3% (Figure 2B). The mean epg per flock was 17.5 (± 33.9), with values between 0 and 154 epg throughout all the study. Among the positive flocks, 21% carried a medium or high infection level with a mean egp higher than 40. For the six years of the study, we found significant differences (p<0.01) in the epg between 2007, with the highest values (38.2 ± 55.9), and 2011, with the lowest number of eggs in faeces (2.1 ± 4.1) (Figure 3B).

**Figure 6:**
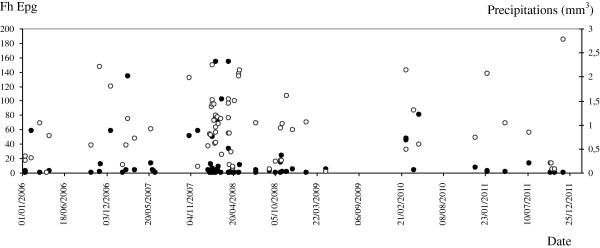
**Distribution of the *Fasciola hepatica *eggs per gram (epg) in faeces along the six years of the study (black spots) in comparison to the precipitations (mm**^**3**^**) (white spots).**

When relating the *F. hepatica* epg to the climate data, it can be observed that the values were significant correlated (p<0.05) with the minimum humidity and precipitations, and, also influenced by the average humidity (Table 2). The distribution of the *F. hepatica* epg in faeces along the six years of the study in comparison to the precipitations, the climate variable with which the highest correlations were shown, is represented in Figure 6.

Only climate data of the León province from October to May for 2006–2011 were analysed to compare the present study with the results obtained by Ferre *et al.*[4] in the same area between October 1992 and May 1993. The data obtained for our sampling period was: average temperature, 9.4°C; maximum temperature, 16.8°C; minimum temperature, 2.8°C; monthly precipitations, 39.9 mm^3^; average humidity, 75.9%. The data obtained for the sampling period in the study of Ferre *et al.*[4] were: average temperature, 11.4°C; maximum temperature, 15.8°C; minimum temperature, 5.9°C; monthly precipitations, 26.2 mm^3^; average humidity, 66.4%.

### Correlations between the climate data

The Pearson’s correlations between the climate data were highly significant between temperature and solar radiation (r = 0.838; p = 0.00) although significantly inverse with humidity (r = −0.738; p = 0.00). The association of solar radiation with humidity was also significantly inverse (r = −0.923; p = 0.00). Precipitations showed a significant relationship with humidity (r = 0.417; p = 0.000) and inverse with solar radiation (r = −0.385; p = 0.000).

## Discussion

In the current study we have analysed the prevalence of helminth infections caused by GIN and the liver fluke *F. hepatica* in sheep flocks of some provinces of the Autonomous Community of Castilla y León for six years, from 2006 to 2011.

For these six years the infection by GIN was diagnosed in all sampled flocks. When we correlated this prevalence with climatic conditions we found that GIN epg values were directly influenced by humidity, principally by the maximum humidity. It is note worthy that in 2008, the year with the highest mean epg of GIN (237.5 epg), matched up with the highest average and maximum humidity values, 78.5 and 95.3%, respectively (Figure 4). Temperature and humidity act to determine the success rate and speed of development of free-living stages, both of which are major influences on the epidemiology of GIN. However, we did not show this association with temperature probably because of the diversity of GIN species found, each one with its own optimal range of temperature to develop. Parasite development rate increases with temperature and while laboratory studies indicate this is linear some recent studies indicate that this may be nonlinear and would have an important impact on the infective stages [8]. According to Dobson *et al.*[9], global warming would decrease the hypobiotic larvae and thus expand the period of the year during which the free-living stages would be active. The consequence is an increase in the rates of ingestion of larval stages by the hosts and also in the infection levels. Rainfall distribution, pasture conditions and soil moisture are more precise determinants than precipitations for the development of the free-living stages. In this context, Besier and Dunsmore [10] concluded that the visual assessment of pasture greenness and the rainfall distribution after the egg deposition were more critical for the development of *H. contortus* than total rainfall and mean evaporation.

Regarding solar radiation, we found that the lowest values were described in 2007 and 2008, with daily mean values of 11.1 and 11.7 MJ/m^2^, respectively, and again tallying with the periods with the highest epg. Solar radiation is related with the number of sunshine hours and in the area of study the lowest values are described between November and February, when the highest GIN epg values were found (Figure 5). The increase in epg in late winter-early spring, associated with the periparturient rise or with the resumption of the development of the arrested larvae, has been previously described in the province of León by Martínez-González *et al.*[5]. These authors also described a second increase in egg count in mid summer that might have resulted from larvae which had hatched from eggs deposited in June-July, when the conditions of temperature and humidity were sufficient to lead infected larvae to migrate to the herbage. However, this second peak does not always happen and it depends on the climatic conditions of each year.

Comparing the current prevalence data in the province of León with a study carried out in the same area during 1991–1992 [5], it can be observed that the current mean epg is slightly higher (286.7 ± 426.7; range: 5–2,552 epg) than for those years (134.3; range: 0–2,050 epg) with a possible cause being the differences in climatic conditions depending on the sampling year. When comparing the climate data registered during 1991–1992 and the period 2006–2011 (Table 3) we observed that the current average temperature is 0.32°C lower (11.93°C) although the maximum temperature is 0.45°C higher (17°C) and the minimum one is 0.5°C lower (5.2°C); the annual rainfalls are very similar between both periods of time despite the fact that the humidity is currently 3.38% higher. According to EEA [11], the climate in Europe has warmed more than the global average, with a 0.95°C increase since 1900. Moreover, temperatures in winter have increased more than in summer and the warming has been greatest in NW of Russia and the Iberian Peninsula.

The latitude of the area of study is above 40° and is characterized by a Mediterranean climate with continental and Atlantic influences. The GIN species found in this study were the same as other authors described previously in the same location and in the neighbouring Autonomous Community of Galicia (NW of Spain) [12,13]. In this latitude *Teladorsagia* and *Trichostrongylus* remain the dominant genera [14] although we found that *Teladorsagia circumcincta* was the most prevalent, in agreement with Álvarez-Sánchez *et al.*[12] who described this species as the most frequent (48%) in the same area of study.

In relation to fasciolosis, its prevalence in flocks from Castilla y León was 59.3%, however, in the province of León the prevalence was 60.5%, which is significantly higher than the percentages described between October 1992 and May 1993, 26.7% (mean 10 epg; 5–450 epg) [4]. A study carried out by Manga *et al.*[15] in this province between 1986 and 1987 also reported very low prevalence values of 14.67% per sheep. This important rise in fasciolosis at the present time could be determined by a change in climatic conditions or it could be also favoured by other factors such as an increment of the irrigated areas in the Autonomous Community and/or due to the development of anthelmintic resistance (AR) [16]. The infection produced by *F. hepatica* has not reached the level of AR as in nematodes [17] although some case has already been described in this area of study [18]. Because of this reason we analysed the climatic conditions during the months of the study of Ferre *et al.*[4] in the province of León and compared their data with ours during the same sampling months. It is worth noting that although the current average temperature (9.4°C) is lower than 20 years ago (11.4°C), the reason is because of the present minimum temperature has decreased although the maximum one is 1°C above. On the other hand, the percentage of humidity nowadays is increased, and, according to Vara del Río [16], presumably by the presence of irrigated areas to encourage new crops in the province. As it is shown in the map (Figure 1), all sampled flocks were located in towns with irrigated areas or near rivers, which is essential for the development of fasciolosis. In general, since the 1990s the government has funded the installation of modern irrigation systems to encourage new crops in the Autonomous Community of Castilla y León. Therefore, it is possible that that the man-made modifications of the environment have favoured significantly the increasing humidity and accordingly it also has influenced the epidemiology of the infection. A similar fact was described by Esteban *et al.*[19] in the Peruvian Altiplano, who pointed out that the quick lymnaeid colonisation of an artificial irrigated area gave rise to a high human hyperendemic area. Higher values of maximum temperatures and humidity could have permitted a better development of the intermediate stages of *F. hepatica* leading to an increase of the prevalence nowadays. Mas-Coma *et al.*[20] reviewed how the temperature has a direct and pronounced effect on the production of cercariae in the intermediate molluscan hosts, however, we did not find this association. The reason could be the short period of time, one month, which was analysed to relate the climatic conditions with the prevalence data bearing in mind that the life cycle of *F. hepatica* outside its definitive host can take several months. According to our results, the infection prevalence was related with the minimum humidity and precipitation values. It has been stated that relative humidity between 70 and 100% is necessary for the reproduction of the intermediate host snails of *F. hepatica*; furthermore, temperatures around 10°C are required for egg hatching and the release of cercariae from the intermediate snail host [21].

## Conclusions

After analyzing the effect of climatic conditions on the helminth infection level for the period 2006–2011 we found that the prevalence of GIN and *F. hepatica* infections were directly influenced by the humidity and also by precipitations in case of *F. hepatica*. Comparing the current prevalence with studies carried out in the same area in the early 1990s, we observed that currently the mean epg of GIN is slightly higher with a possible cause being the differences in climatic conditions depending on the sampling year. Regarding *F. hepatica* infection, its prevalence rose significantly probably favoured by an increase in irrigated areas in the area of study.

## Competing interests

The authors declare that they have no competing interests.

## Authors’ contributions

MMV assisted with experimental design, conducted the experiments, analysed the data, interpreted data and drafted the initial manuscript. DRP, JMMP, CCP, MRF, NFP, CGL and LCO conducted the experiments and reviewed the manuscript prior to submission. FARV assisted with experimental design, data interpretation, offered general expertise and advice and produced the final version of the manuscript. All authors approved the final version of the manuscript.
